# 2116. Long-term (2-year) outcomes and complications of COVID-19 in solid organ transplant (SOT) recipients

**DOI:** 10.1093/ofid/ofac492.1737

**Published:** 2022-12-15

**Authors:** Daniel Burack, Marcus R Pereira, Elizabeth Verna

**Affiliations:** NYP-Columbia University Medical Center, New York, New York; NYP-Columbia University Medical Center, New York, New York; NYP-Columbia University Medical Center, New York, New York

## Abstract

**Background:**

The long-term complications of COVID-19 infection in the general population include mortality, re-infection, secondary infection, persistent organ dysfunction, and symptoms of long-COVID. The prevalence of these outcomes and impact on graft function in solid organ transplant (SOT) remain uncertain. We aim to describe these complications in a large series of SOT with COVID-19 with 2 years of long-term follow up.

**Methods:**

We retrospectively studied all adult (age >18) SOT from a single center hospitalized with SARS-CoV-2 diagnosed by nasopharyngeal swab between 3/10-5/30/2020. Patients with early mortality within 28 days were excluded. Outcomes including mortality, allograft rejection, allograft failure, secondary infections, COVID-19 re-infections, post-COVID complications (oxygen requirement, chronic renal or cardiac dysfunction), and symptoms of long-COVID were analyzed. Re-infections were characterized by severity and likely variant based on local variant predominance.

**Results:**

117 SOT recipients were hospitalized with COVID-19 in the study period. 94 survived the first 28 days and were followed for a median of 751 (742-760) days post-infection. 9 (9.57%) died within 1 year of infection and 14 (14.9%) within 2 years. 21 (22.3%) had ≥1 episode of allograft rejection and 21 (22.3%) had allograft failure. 11 (9.4%) were re-infected with COVID-19 at a median of 603 (389-642) days following initial infection, of whom 2 (18.2%) were hospitalized and 0 died. 43 (45.7%) had secondary infections and 18 (19.1%) with multi-drug resistant organisms. 32 (34.0%) developed new chronic kidney disease or end-stage renal disease, 25 (26.6%) had new cardiovascular disease, and 8 (8.51%) had a prolonged oxygen requirement following infection. Of reported long-COVID symptoms, fatigue (26, 27.7%), dyspnea (18, 19.1%), and cough (11, 11.7%) predominated with 25 (26.6%) having >1 symptom.

**Figure:**
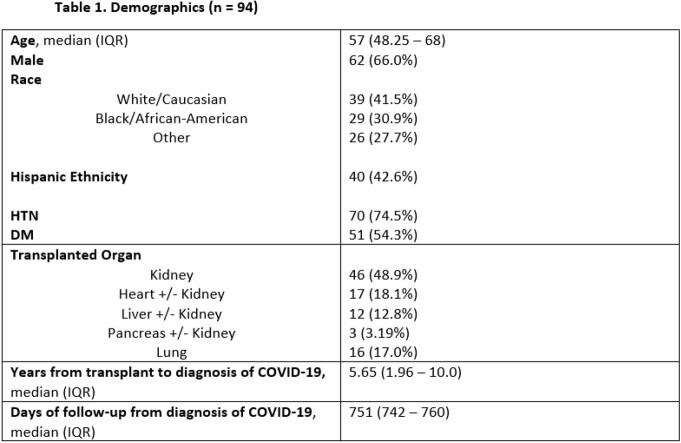

**Figure:**
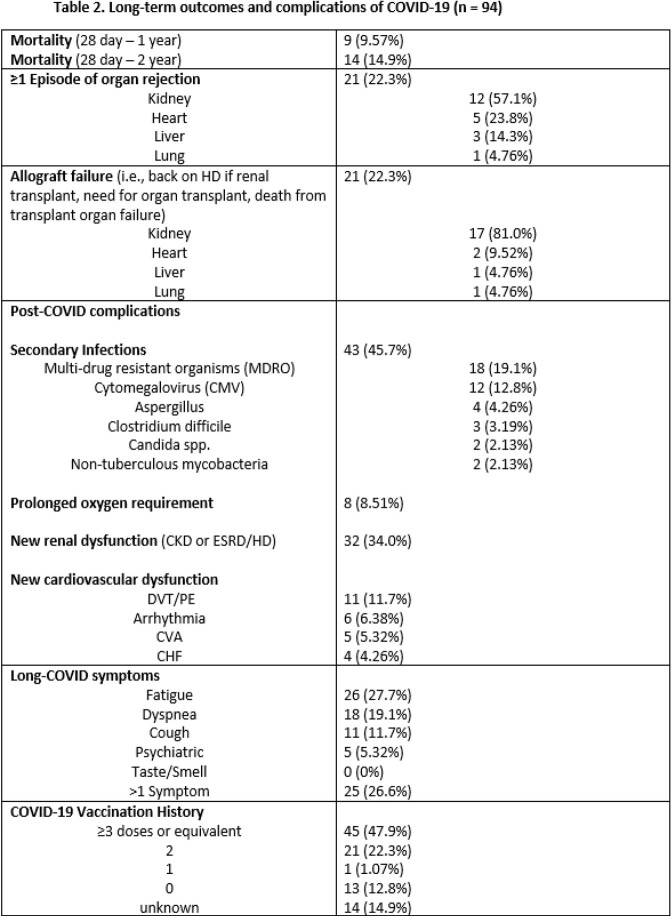

**Figure:**
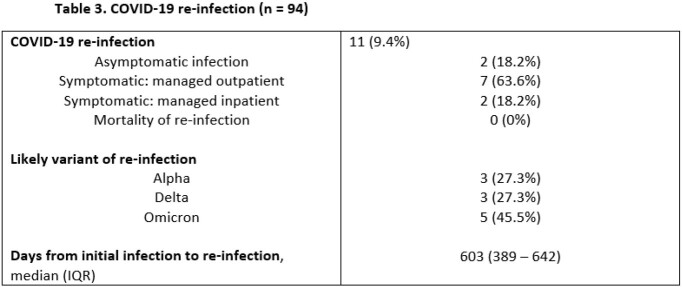

**Conclusion:**

In this large cohort of SOT recipients hospitalized during the first wave of the COVID-19 pandemic, long-term 2-year follow-up showed high rates of mortality, allograft rejection, allograft failure, secondary infection, organ dysfunction, and symptoms consistent with long-COVID. Ongoing study of the impact of these complications will be crucial to improving outcomes in SOT recipients.

**Disclosures:**

**Marcus R. Pereira, MD**, Hologic: Grant/Research Support|Merck: Grant/Research Support|Moderna: Grant/Research Support|Shire/Takeda: Grant/Research Support|Takeda: Advisor/Consultant|Union Therapeutics: Advisor/Consultant **Elizabeth Verna, MD**, Salix: Grant/Research Support.

